# Impact of Sm^3+^ Ions on Oxygen Vacancy Formation in Ceria Systems

**DOI:** 10.3390/molecules30234615

**Published:** 2025-12-01

**Authors:** Masoomeh Keyhanian, Iskra Z. Koleva, Hristiyan A. Aleksandrov

**Affiliations:** Faculty of Chemistry and Pharmacy, Sofia University “St. Kliment Ohridski”, 1164 Sofia, Bulgaria; mkeyhanian@chem.uni-sofia.bg

**Keywords:** density functional theory, ceria, Sm dopant, reducibility, oxygen vacancy formation energy

## Abstract

DFT calculations have been employed to investigate the impact of Sm dopant(s) on the structure and stability of periodic CeO_2_(111) slab and Ce_140_O_280_ nanoparticle systems. We found that substituting Ce ion(s) with Sm ion(s) in the surface layer yields the most favorable doped configurations in both systems. Further, we evaluated how Sm ion(s) modify the reducibility of the systems—a key process in the catalytic applications of ceria. It has been found that a substantial reduction in oxygen vacancy formation energy occurs due to the presence of Sm ion(s), with values of 1.24 and 0.29 eV for mono- and bi-doped CeO_2_(111), respectively, which are considerably lower than 2.43 eV obtained for the pristine ceria slab. As the system changes from slab to nanoparticle, mono-doping reduces this energy to 0.25 eV—about four times lower than that calculated for the pristine Ce_140_O_280_ nanoparticle. The presence of a second Sm ion within the nanoparticle leads to a dramatic decrease in E_vac_, making the reduction process exothermic. In either slab or nanoparticle models, the Sm^3+^ ions prefer to be in close proximity to each other, and the formation of oxygen vacancies is most energetically favorable in the vicinity of Sm^3+^ ions.

## 1. Introduction

Metal oxides possess diverse properties and behaviors due to the extensive variety in their composition, as well as electronic and geometric structures, which can give rise to specific functionalities and chemical activities [1,2]. Among them, cerium dioxide (ceria, CeO_2_) nanomaterials have attracted significant attention owing to their unique combination of high ionic conductivity [3], excellent redox properties, and good thermal and chemical stability, all of which are influenced by factors such as size, shape, crystallinity, oxidation states, dopant, and structural or surface defects [4]. These features make them highly valuable for a wide range of applications in catalysis, energy, environmental, and biomedical fields [5,6,7,8,9,10].

In oxidation reactions, particularly those proceeding via the Mars–van Krevelen mechanism, the reducibility of oxide catalysts is a key feature [11]. A common descriptor of oxide reducibility is the energy required to create an oxygen vacancy, which reflects the tendency of the oxide to release lattice oxygen or transfer it to an adsorbed species [12]. Among reducible oxides, ceria-based materials stand out for their distinctive catalytic properties, which stem from their ability to undergo facile redox transformation between the Ce^4+^ and Ce^3+^ oxidation states [13,14]. This redox activity can be initiated in two main ways. One involves direct electron transfer from an adsorbed metal or molecular species to the ceria surface [14], resulting in partially cationic adsorbates—an effect that is particularly significant when working with metals supported on ceria systems. The other involves the creation of an oxygen vacancy, where the release of a lattice oxygen atom leads to the localization of two electrons on Ce cations, which are consequently reduced to Ce^3+^ (Ce 4f^1^) [15,16]. The coexistence of Ce^3+^/Ce^4+^ states and the associated oxygen vacancies not only contribute to ceria’s high catalytic reactivity in both oxidation and reduction reactions but also enhance its ionic conductivity, making it highly suitable for applications such as solid oxide fuel cells and oxygen sensors [17,18,19].

With growing interest in improving the reducibility and catalytic performance of ceria-based materials, extensive efforts have been devoted to various approaches [20,21,22,23,24,25], among which doping ceria with heteroatoms has proven to be a powerful strategy in modifying its electronic structure [26,27]. Experimental and computational studies have explored the impact of both metal dopants [28,29,30,31]—such as noble metals, rare earth elements, or transition metals—and nonmetal dopants [32,33,34], such as C and N, on the electronic structure of CeO_2_ materials. In a comparative experimental study of CO catalytic oxidation over ceria doped with Zr^4+^ and Hf^4+^, it was found that incorporating the smaller Hf^4+^ cation into the ceria lattice was more effective in promoting the formation of oxygen vacancies, which in turn enhanced the reducibility of composite [35], consistent with the theoretical studies conducted by some of us [36,37]. Studies have also shown that doping ceria with lower valent cations, like Y^3+^, effectively creates oxygen vacancies [38,39,40,41]. The nature of changes caused by dopants strongly depends on the valence state of the substituting cation relative to the host cerium ion (Ce^4+^). When a dopant possesses the same valence as Ce^4+^ ion, the difference in ionic radii between the dopant and Ce^4+^ can lead to structural distortions, which can alter the strength of the Ce-O and dopant-O bonds. In contrast, aliovalent doping, where Ce^4+^ is substituted by a cation of different valence, is more complex, as it creates a charge imbalance that must be compensated through the formation of additional structural defects to preserve overall charge neutrality, and consequently leads to notable catalytic performance in oxidation reactions compared to isovalent-doped ceria.

Among the elements commonly used as dopants for ceria, samarium has attracted particular interest. Yang et al. [42] synthesized a series of Sm-doped ceria catalysts with varying doping levels via the hydrothermal method and evaluated their catalytic activity for CO oxidation. They reported that the morphology and structural properties of the catalysts depended on the percentage of dopant, which affected their catalytic performance. The results revealed that Sm-doped ceria had enhanced potential for CO oxidation compared to undoped ceria. A study by Chanapattharapol et al. also investigated how adding Sm into ceria-based support would affect its performance as a catalyst [43]. The results showed that incorporating Sm into the material enhanced its structural and chemical properties, leading to improved catalytic activity for the water gas shift reaction compared to undoped ceria. Anjaneya et al. [44] had experimentally studied the structural, morphological and electrical properties of Ce_0.8_Ln_0.2_O_2−δ_, (Ln = Y^3+^, Gd^3+^, Sm^3+^, Nd^3+^ and La^3+^) solid solutions and concluded that Sm-doped ceria exhibits the highest oxygen ionic conductivity among the all studied doped ceria due to the proximity of the ionic radius of Sm^3+^ to the so-called critical radius, which for trivalent cation in ceria is 103.8 pm [45].

In Sm-doped ceria systems, the typical and energetically favorable doping mechanism, especially at lower concentrations of the dopant (below 7%), is the substitution of Ce cations by Sm, rather than the occupation of interstitial sites. Forcing the Sm dopant to occupy interstitial positions in the fluorite structure of CeO_2_ will cause a large local distortion due to the relatively large atomic radius of Sm^3+^. In support of this statement is the research of Chen et al. [46].

Based on the preceding discussion, previous studies have primarily focused on the effects of Sm doping on structure and catalytic activity from an experimental perspective, while key questions about the changes in surface configuration, electronic properties, oxidation states, and reduction behavior of ceria surfaces after Sm doping remain unanswered. To address these ambiguities, we conducted a study based on DFT calculations, similar to those reported for Zr- and Hf-doped ceria [36,37], aiming to provide valuable insights and clarify relevant characteristics. In the present study, we employed CeO_2_(111) slab and Ce_140_O_280_ nanoparticle ceria models to analyze the energetic stabilities and structural changes of Sm-doped ceria with various Sm dopant orderings. This allows us to determine the preferred substitution site of Sm dopants by assessing their stability in the surface or subsurface layers of ceria systems. Next, the impact of Sm^3+^ cations on the reducibility of ceria was evaluated by calculating the oxygen vacancy formation energy for selected Sm-doped ceria models, which are then compared with those of pristine ceria models as the reference systems.

In the current study, we are focused on pristine regular CeO_2_(111) surface and small Ce_140_O_280_ nanoparticle models to explore the influence of the Sm^3+^ dopant on the formation of oxygen vacancies in ceria systems. However, we note that depending on the experimental conditions, which can include water vapor and/or CO_2_ in the gas phase, various species such as hydroxyl groups, carbonate and formate species, etc., may exist on the ceria surface [47,48,49]. These species may influence the reactivity and catalytic behavior of the Sm-doped ceria systems.

## 2. Results and Discussion

### 2.1. Sm-Doped CeO_2_(111) Surface

#### 2.1.1. Sm Mono- and Bi-Doped Surfaces

**Figure 1:**
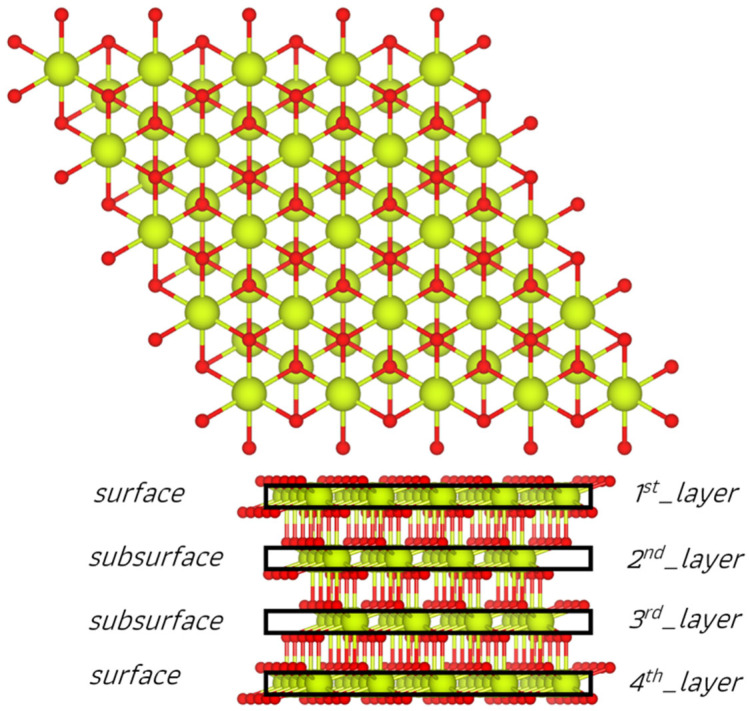
Top and side views of the ceria slab model with the stoichiometry Ce_64_O_128_. The bold black rectangles indicate the type of Ce^4+^ cations and their layers. Yellow and red spheres represent cerium and oxygen centers, respectively.

The model employed is a four tri-layer slab containing Ce^4+^ cations, which can be categorized into two distinct types: surface and subsurface. The 1st and 4th layers constitute the surface, while the 2nd and 3rd layers form the subsurface region, as shown in Figure 1. These numbers are used below to distinguish the corresponding surface layers and to name the Sm-doped structures. The model provides two distinct sites for mono-doping with Sm (one Sm^3+^ ion per unit cell): one on the surface, Surf, and one in the subsurface area, Sub. To identify the most suitable sites for bi-doping (two Sm^3+^ ions per unit cell), the substitution of two Ce^4+^ with two Sm^3+^ ions was explored by considering different configurations based on whether the replaced Ce^4+^ ions were both at the surface (11, 14), both in the subsurface layers (22, 23), or one at the surface and the other in the subsurface region (12, 13). Furthermore, the impact of the distance between the two Sm atoms on the stability of the models was also analyzed by considering whether they were located close to each other or far apart, which were labeled by the capital letters C and F, respectively. Combining these two criteria led to a total of 12 different models for analysis of Sm bi-doped ceria surface, with each model indicated by using two digits denoting the layers where both Sm^3+^ cations are positioned, followed by capital letters “C” or “F” showing whether Sm^3+^ cations are close or far from each other (Table 1).

For Sm mono-doped ceria, surface layer substitution is energetically preferred by 0.09 eV over subsurface substitution, as reported by the relative energies in Table 1. In the case of Sm bi-doped ceria, the lowest energy configuration is 11_C, where the two Sm ions are adjacent on the surface. The next three models—11_F, 14_C, and 14_F—have identical energies and are only 0.07 eV less stable than 11_C, whereas 22_F and 23_F are the least stable and higher in energy by 0.25 eV compared to the most stable configuration. Our results generally indicate that the foreign Sm ion(s) preferably occupy low-coordinated positions in the ceria lattice. Moreover, while placing Sm ions close to each other leads to more stable bi-doped ceria structures in most models, the stability of substitutions in the 13 and 14 layers of CeO_2_(111) is not affected by the Sm-Sm distance. In all the cases where Sm^3+^ cations are placed on next neighboring positions, i.e., with Sm-Sm distance of ~385 pm, the structures are more stable by 0.07–0.12 eV than the corresponding structures where Sm^3+^ cations are far from each other with Sm-Sm distances of 670–866 pm. In the situations where the closest Sm-Sm distances are larger than 670 pm, the stability of the corresponding “C” and “F” structures is essentially the same.

Following the investigation of the stability of models, we analyzed the magnetic moments of Ce, Sm, and O ions to answer the question of whether Sm mono- and bi-doping of the ceria surface led to isovalent or aliovalent substitution. Individual ion magnetization analysis revealed that the magnetic moment of Sm ion(s) in both doping cases is ~5.00 µB, which confirms the presence of Sm^3+^ cation(s). Since no magnetic moment was detected for any of the Ce ions after Sm doping, the substitution of Sm^3+^ with Ce^4+^ indicates an aliovalent process. Regarding charge compensation, further analysis of Ce and O magnetization revealed that charge neutrality in these systems is balanced through neighboring partially oxidized O centers near the Sm ion(s) with the magnetic moments in the range of −0.10 to −0.30 µB.

#### 2.1.2. Reducibility of Surfaces with Sm Mono- and Bi-Doping

**Figure 2:**
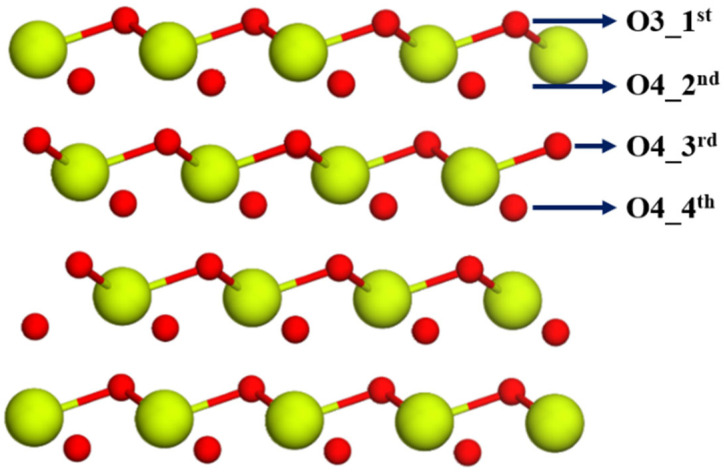
Side view of the O-atom positions on the stoichiometric CeO_2_(111) surface, highlighting the sites for oxygen removal. Each label is followed by two numbers: the first indicates the coordination (three- or four-coordinated) of the O center to be removed, while the second, separated by an underline, refers to the corresponding anionic layer in the stoichiometric structure. The color scheme is the same as in Figure 1.

After identifying the most stable Sm-doped CeO_2_(111) surface structures, it is important to investigate how the presence of Sm dopant(s) influences the reducibility of ceria, specifically whether oxygen vacancy (O_vac_) generation is hindered or facilitated. For reference, our initial focus was on the pristine ceria slab model. As shown in Figure 2, there are four nonequivalent O positions in our slab model located in the four O layers of the surface and subsurface CeO_2_ tri-layers. Based on this, we created four structures with O vacancies, as in each case, one nonequivalent O center of every type is removed. According to Table 2, O vacancy formation in the undoped system, which involves the reduction of two Ce^4+^ (4f0) cations to Ce^3+^ (4f1), requires energies between 2.43 and 3.25 eV. The most energetically favorable structure resulted from the removal of a four-coordinated O center located in the second anionic layer of the stoichiometric structure. Compared to our previous undoped ceria slab model [41], which yielded O vacancy formation energies of 2.55–2.88 eV down to the third anionic layer, our current results indicate similar stability orders for the O_vac_ sites, with slight discrepancies that can be attributed to the different slab geometry. Subsequently, for the Surf and Sub models of Ce_63_SmO_128_, the reducibility of the Sm mono-doped CeO_2_(111) slab was explored using 6 and 8 Ce_63_SmO_127_ structures, respectively, with the detailed results in the ESI. For these models, oxygen vacancies were generated down to the third anionic layer below the position of the Sm dopant in the ceria slab. This allowed us to determine the influence of the Sm ion on reducibility by changing the distance of the removed oxygen from the Sm site, in both parallel and perpendicular directions. Analyzing O vacancy formation energies at various sites of the Sm mono-doped ceria slab models revealed distinct preferences. In both Surf and Sub models, vacancy formation by substituting Sm by Ce was more favorable, with energy ranges of 1.41–1.90 and 1.24–2.04 eV, respectively. When the doping site is located in the subsurface of the ceria framework, the most suitable O removal occurs from a four-coordinated O center in the second anionic layer—the same layer that exhibits the lowest O vacancy formation energy in the pristine CeO_2_(111) slab—close to the Sm dopant. In contrast, the O removal from the fourth anionic layer, far from the subsurface dopant site, resulted in the least energetically favorable configuration. A similar pattern was observed for the Surf model of Ce_63_SmO_128_, where the O4_2nd and O4_3rd configurations emerge as the most and least stable structures, respectively, upon O removal. In the former case, the removed O site can lie far or close to the dopant site as the stability of both corresponding structures, O4_2nd_far and O4_2nd_close is within 0.04 eV. Evaluation of the magnetic moments in the Sm mono-doped systems revealed that regardless of the vacancy position, creating an O vacancy in different Ce_63_SmO_128_ models triggers charge redistribution that stabilizes the −2 oxidation state of particular O atoms close to Sm^3+^ ion and simultaneously leads to the reduction of Ce^4+^ to Ce^3+^ in the vicinity of the vacancy. In both most favorable sites of the Surf and Sub models, the obtained Ce^3+^ ion sits on the surface layer near the vacancy site. However, in the Surf model, the Ce^3+^ ion is located far from the Sm dopant, while in the Sub model, it lies directly above and adjacent to the Sm3+ ion and the removed four-coordinated O site.

To determine the most probable oxygen vacancy site in the Sm bi-doped CeO_2_(111), we analyzed three models of Ce_62_Sm_2_O_128_. Among these, 11_C and 11_F correspond to surface doping and are the most stable, while the third one, 22_C model, is the most stable configuration among the four subsurface-doped models. Considering the full range of O vacancy formation energies, the introduction of two Sm^3+^ dopants makes vacancy formation notably more favorable than one Sm^3+^ substitution, as the energy values in the former case are lower by ~1 eV than in the latter one. The most favorable configuration, O4_2nd_C, features Sm^3+^ ions located in the first subsurface layer and shows a vacancy formation energy of 0.29 eV, which is ~8 and ~4 times lower than that of the most stable structures with an O vacancy in the pristine slab model, O4_2nd, and Sm mono-doped slab model, O4_2nd_C. Even the least favorable vacancy of the modeled Ce_62_Sm_2_O_127_ structures, a four-coordinated oxygen removal from the fourth anionic layer, yields E_vac_ value of 0.98 eV, which remains more favorable than the most stable Ce_63_SmO_127_ structure. Focusing on the surface-doped models, 11_C and 11_F, the preferred O vacancy is slightly less energetically favorable by 0.21 and 0.16 eV, respectively, than the most stable structure of 22_C model. In the 11_F model, the vacancy forms by removing a four-coordinated O atom from the second anionic layer, a pattern also observed in the Surf and Sub models of Ce_63_SmO_127_ as well as for the pristine slab model. On the contrary, a three-coordinated oxygen atom from the first anionic layer appears to be the most preferential vacancy site in the 11_C model, requiring 0.50 eV. Magnetic moment analysis of individual atomic centers in all reduced Sm bi-doped ceria configurations with one introduced O vacancy revealed charge compensation, which re-established the −2 oxidation state for all O centers, as in the pristine ceria system. It is important to note that while the potential for Sm^2+^ formation was also explored by us for the O vacancy structures, our calculations demonstrate no evidence of its presence.

To summarize, Sm doping significantly affects O vacancy formation energies. Our comparison of the most stable O vacancy locations in both mono- and bi-doped CeO_2_(111) surface suggests a preference for vacancy creation in the second anionic layer of the doped ceria surface model.

### 2.2. Sm-Doped Ce_140_O_280_ Nanoparticle

#### 2.2.1. Sm Mono- and Bi-Doped Nanoparticle

**Figure 3:**
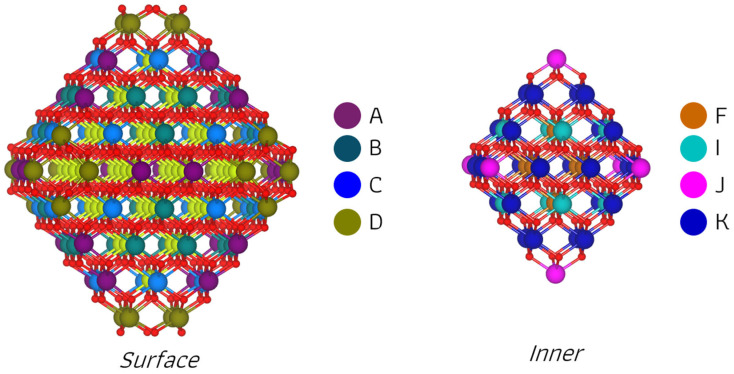
Structure of Ce_140_O_280_ nanoparticle. In the full structure (**left**), non-equivalent surface Ce^4+^ cations are marked with different colors, while inner Ce^4+^ cations are shown in yellow. For better visual understanding, the core of the nanoparticle is presented separately on the (**right**), where non-equivalent inner Ce^4+^ cations are also distinguished by specific colors.

With reference to our earlier work [37], where the description and structural features of the Ce_140_O_280_ nanoparticle were discussed in detail, eight different types of Ce^4+^ cations have been identified: four located on the surface and four within the inner (subsurface) region, as highlighted in Figure 3. Accordingly, we have eight modeling options for Sm mono-doped nanoparticles, each created by substituting one Ce^4+^ with Sm^3+^. After relaxation, the stability of these doped NPs was assessed using relative energies, with the results summarized in Table 3. The models were labeled using the S_A, S_B, S_C, S_D, I_F, I_I, I_J, and I_K notations, where the prefix distinguishes surface (S) from inner (I) positions of the nanoparticle, and the following part identifies the particular site shown in Figure 3. The results reveal that although significant changes in the relative energies of the doped NPs are not observed, the S_D position, an edge site between two (111) and also being part of the CeO_2_(100) facet, represents the most favorable location for Sm doping, followed in stability by the I_J position, which places Sm^3+^ in the first subsurface layer just below the (100) facet and is only 0.04 eV less stable, a negligible difference. The inner positions I_F and I_I are identified as the least stable doping sites, located in the first and second subsurface layers, respectively, and bonded to four-coordinated oxygen anions. Excluding the I_J position, the Sm^3+^ cation dopant shows a preference for low-coordinated surface sites, a tendency also observed for Sm mono-doping of the CeO_2_(111) slab, which suggests that the presence of Sm^3+^ in subsurface sites, characterized by a coordination number of 8, results in less stable doped structures compared to those at the surface.

The specific positions S_D and I_J, the two most energetically favorable sites, were selected as target sites for substitution of a second Sm ion to form bi-doped nanoparticles. By considering both close and far distances between the dopants, we modeled four different bi-doped structures, namely DD_C, DD_F, DJ_C, and DJ_F, for subsequent investigations. Our observation from the most stable models, DD_C and DJ_C, indicates a clear preference for the second Sm^3+^ cation to occupy the D site in proximity to the first Sm dopant, resulting in enhanced stability compared to the corresponding models with larger distances, i.e., DD_F and DJ_F (Table 3). Furthermore, the structures with close Sm^3+^ ions on the surface demonstrate higher stability than those with one Sm ion on the surface and the other at an inner site, with an energy difference of 0.07 eV. Structural similarities emerge when comparing our nanoparticle models to the Sm bi-doped CeO_2_(111) slab models. Referring again to Table 1, it is evident that models 11_C, 11_F, 12_C, and 12_F are the most similar structures to our Ce_138_Sm_2_O_280_ nanoparticle models in terms of the positions of Sm^3+^ cations on the surface or subsurface regions as well as dopant-dopant distances. The latter one is valid especially for the 11_C and 12_C structures. Importantly, slight stability differences of 0.08 eV between 11_C and 12_C, and between 11_F and 12_F in favor of the structures with surface location of Sm^3+^ cations are consistent with observations from the nanoparticle models. The most probable reason for Sm^3+^ cations to occupy less sterically hindered surface positions on ceria slab and nanoparticle models arises from their larger ionic radius (107.9 pm) [50] compared to the substituted Ce^4+^ (97 pm) [50], which mitigates lattice strain.

Based on the total magnetization and magnetic moment per atom, it is found that Sm ions adopt a +3 oxidation state when they aliovalently replace Ce^4+^ cations in both mono- and bi-doped ceria nanoparticles, consistent with observations in CeO_2_(111) slab models. To compensate for the local charge imbalance in the mono-doped systems, electron density is taken from the O centers in the first coordination shell of the Sm cation. As a general trend in most systems, more electron density is donated by the O centers with lower coordination numbers. For instance, the surface models of A, B, and C reveal a partial reduction in electron density mainly on the three-coordinated oxygen ions directly bonded to the dopant. In the most stable S_D model, the two-coordinated oxygen atoms directly bonded to the dopant exhibit the largest partial electron depletion. Regarding the inner models of I, F, J, and K, where Sm^3+^ was located in the first and second subsurface layers of the nanoparticle, the partial electron depletion is found only on the four-coordinated O atoms bonded to the dopant. Interestingly, a broader electron distribution over all eight next-nearest neighbor oxygen ions coordinated to Sm^3+^ is observed only in the case of I_J model. In the four models of Ce_138_Sm_2_O_280_ system, the highest electron depletion is again concentrated on the two-coordinated oxygen ions, although they are located at relatively large distances from both dopants.

#### 2.2.2. Reducibility of Nanoparticles with Sm Mono- and Bi-Doping

**Figure 4:**
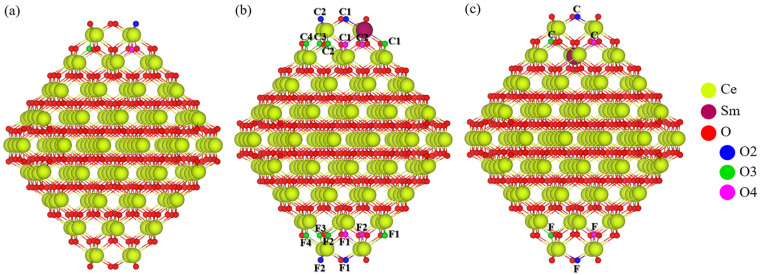
Optimized structures and O ion positions examined in the pristine structure (**a**) and selected Sm mono-doped nanoparticles, S_D (**b**) and I_J (**c**), for oxygen removal. Each O atom label on the nanoparticles consists of a capital letter indicating its distance from the dopant (C for close, F for far), and for the S_D model only, digits are used to distinguish considered non-equivalent O atoms with the same coordination number. O atoms are color-coded by coordination: blue for two, green for three, and purple for four.

To investigate oxygen vacancy formation in the Sm-doped nanoparticles, we removed an oxygen ion from two-, three-, and four-coordinated sites in six doped models, including the most stable Sm mono-doped structures, S_D and I_J, and all four considered models of the Ce_138_Sm_2_O_279_ system. Before detailing the doped systems, we first analyzed the pristine nanoparticle to provide a reference point for evaluating reducibility after doping. For the pristine Ce_140_O_279_ nanoparticle, the lowest (1.04 eV) and highest (2.22 eV) O vacancy formation energies correspond to the removal of the two- and three-coordinated surface oxygen atoms labeled in Figure 4, respectively (Table S2). Removing a four-coordinated subsurface oxygen resulted in the same vacancy formation energy as removing a two-coordinated oxygen (Table S2), since the two-coordinated oxygen migrates to fill the vacancy created by the removed four-coordinated oxygen during geometry optimization. Consistent with known ceria chemistry, two Ce^4+^ cations were reduced to Ce^3+^ upon vacancy formation; in the O_2_ model of the Ce_140_O_279_ nanoparticle (Table S2), these Ce^3+^ cations are located at the edge D positions (Figure 3), directly adjacent to the removed two-coordinated O center.

Focusing on Sm mono-doping, Figure 4 illustrates that the Sm dopant in the S_D model introduces several nonequivalent O anion sites, which are distinguished not only by two-, three-, and four-coordinated centers but also by their distance from the dopant. To identify these sites, numerical labels were assigned, as shown in Figure 4b. By considering both close and far positions of oxygen ions available for removal, a total of 16 reduced structures were investigated for this mono-doped nanoparticle model. Regarding the Ce_139_SmO_279_ nanoparticle, when the Sm^3+^ occupies the I_J position, the resulting symmetry reduces the number of considered models for O vacancy formation. In this case, six models were investigated, corresponding to the removal of oxygen ions from one of three coordination types located at different distances from the dopant. The calculated O vacancy formation energies indicate that the lowest values for models S_D and I_J are 0.34 and 0.25 eV, respectively—about four times lower than that for the most stable structure of Ce_140_O_279_—and correspond to the removal of a four-coordinated O anion near the Sm^3+^ ion. However, in agreement with the situation observed in the pristine nanoparticle, structural relaxation causes all initially four-coordinated vacancy models to converge to structures equivalent to those derived from removing a two-coordinated oxygen. This suggests that, consistent with the earlier observation of electron depletion at two-coordinated oxygen sites, the removal of such an O ion results in the most stable defect structure in both Sm mono-doped models. While removing a three-coordinated O center yields the highest vacancy formation energies in both models, these values are still lower than the corresponding ones in the pristine ceria nanoparticle. Another important finding for the Ce_139_SmO_279_ models is that O vacancy formation energies are higher for O ions removed from positions far from the dopant than the E_vac_ values for the structures where O vacancies are created close to the dopant. Furthermore, similar to the observations in the Sm mono-doped CeO_2_(111) slab model, spin-polarized calculations demonstrate that forming an O vacancy in the Ce_139_SmO_280_ nanoparticle models induces an unpaired electron on one of the neighboring Ce ions with respect to the vacancy site, indicating the presence of a Ce^3+^ ion in our system. At the same time, specific O anions with partial electron depletion become stabilized and display no atomic magnetic moments.

**Figure 5:**
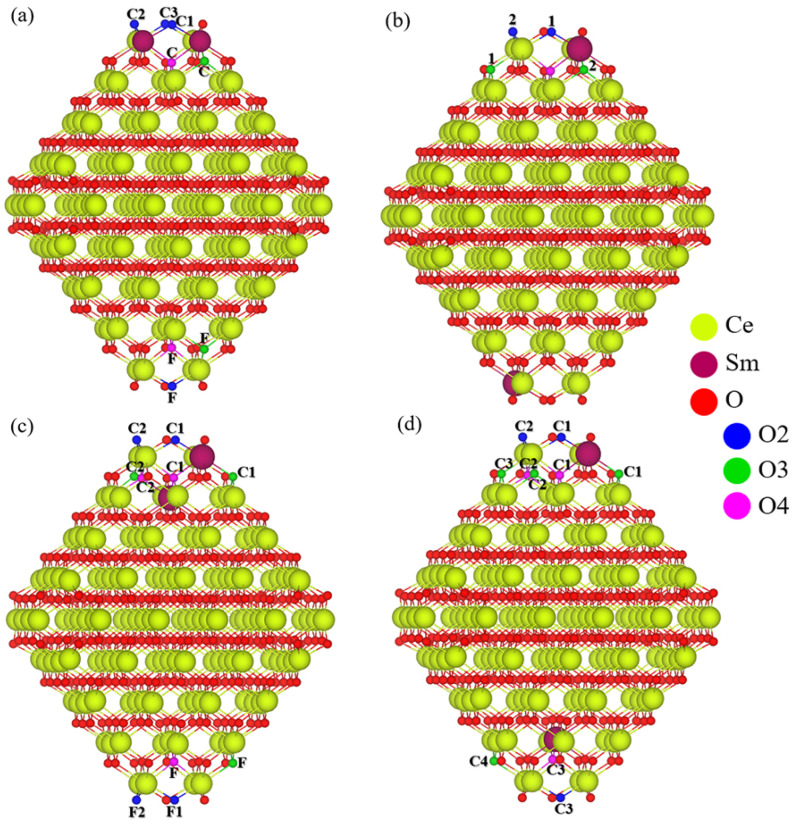
Optimized structures and O ion positions examined in the selected Sm bi-doped nanoparticles, (**a**) DD_C (**b**) DD_F (**c**) DJ_C, and (**d**) DJ_F, for oxygen removal. Each O atom label on the nanoparticles consists of a capital letter indicating its distance from the dopant (C for close, F for far) and digits are used to distinguish considered non-equivalent O atoms with the same coordination number. O atoms are color-coded by coordination: blue for two, green for three, and purple for four.

For the Ce_138_Sm_2_O_280_ system, defective structures were constructed by modeling O anion removal at various positions depending on the dopants location, with 8 removal sites for DD_C, 5 for DD_F, and 10 for each of the two DJ arrangements (Figure 5). Within the models considered, DD_F offered the fewest candidate sites for creation of the O vacancy due to the equivalent positions of Sm ions. Moreover, in both DD_F and DJ_F models, where the two Sm ions are located far from each other, our focus was restricted to the creation of vacancies near the dopant sites. These non-equivalent sites, characterized by the coordination number of the removable O ion, were labeled numerically (Figure 5). According to the oxygen vacancy formation energies presented in Table 4 and Table S2, the vacancy formation at the O2_C1 position of the DJ_C model is an exothermic event with an energy value of −0.20 eV, indicating that such process can occur spontaneously. Next in stability are the O2_C1 site in the DD_C model and the O2_1 site in the DJ_F, both of which are also slightly exothermic and very close in energy. Moreover, while all removal sites in the DD_F model show endothermic vacancy formation energies, the O2_1 site stands out with a value of 0.09 eV, which makes it considerably more stable than the most stable structure in the Ce_139_SmO_279_ system. Overall, these results establish several consistent trends for all considered models of the Ce_137_Sm_2_O_280_ system, in agreement with our earlier observations for pristine and Sm-mono-doped nanoparticle systems. The most and least favorable oxygen removal sites derive from two- and three-coordinated O centers, respectively, and all structures where a four-coordinated O center is initially removed relax to the same final configurations observed when O is removed from a two-coordinated center. For the DD_C and DJ_C models, oxygen vacancy formation energies increase when the removed O and Sm ions are located far from each other compared to configuration where they are close. Now that we have discussed the most and least favorable positions for O removal, it is noteworthy that in each of the four models of the Ce_137_Sm_2_O_280_ system, the two-coordinated O that forms the most favorable vacancy is consistently located adjacent and directly bonded to the Sm ion(s) on the surface at the D site. From the magnetization per ion point of view, two distinct charge compensation mechanisms were identified in the various structures of the Ce_137_Sm_2_O_280_ system, which notably contribute to stabilizing the system. The first mechanism involves Sm ions with five unpaired electrons (i.e., Sm^3+^ ions) and is found in all defective structures of the DJ_F model, which display the minimum energy difference between its most and least stable configurations compared to other models, as well as in all structures with a two-coordinated O vacancy where E_vac_ < 0.54 eV or a three-coordinated O vacancy where E_vac_ < 1.58 eV. The second mechanism, observed in the remaining structures, i.e., two-coordinated O vacancies with E_vac_ ≥ 0.54 eV and three-coordinated O vacancies with E_vac_ ≥ 1.58 eV, involves a more complex charge compensation, including the presence of two Sm^3+^ cations and one Ce^3+^ cation, with some O ions adopting partial electron depletion. Note that, with the exception of the O3_C and O3_1 positions of the DD_C and DD_F models, respectively, the structures with oxygen vacancies located far from the dopants employ the second compensation scheme. In such cases, a Ce^3+^ appears adjacent to the vacancy, depending on the vacancy position.

In general, the reducibility of Ce_140_O_280_ nanoparticle can be enhanced by Sm doping, which significantly decreases the oxygen vacancy formation energy. From our findings, and similar to the pristine nanoparticle, the most favorable site for vacancy formation corresponds to a two-coordinated center located close to the dopant, a trend observed in both mono- and bi-doped nanoparticles.

## 3. Method and Models

### 3.1. Computational Details

The plane-wave based Vienna Ab initio Simulation Package (VASP) [51,52], version 5.4.4, was employed to perform the periodic quantum-chemical calculations in the framework of Density Functional Theory (DFT). The exchange-correlation effects were described using the generalized gradient approximation (GGA) with the Perdew–Wang 1991 (PW91) functional [53,54]. To mitigate the self-interaction error found in standard GGA functionals [55,56,57], all calculations for both stoichiometric and reduced Sm-doped ceria systems were carried out using the GGA+U approach. This method includes a local Coulombic Hubbard U term, the value of which is usually chosen to reproduce experimental results [58,59,60]. The valence configurations used in the chosen pseudopotentials were Sm (5s^2^5p^6^6s^2^4f^6^), Ce (5s^2^5p^6^6s^2^5d^1^4f^1^), and O (2s^2^2p^4^), and, consistent with previous studies, we applied a U value of 4 eV to correct the Ce 4f orbitals [25,61,62]. A cutoff of 415 eV for the kinetic energy was applied to select the plane-wave basis set. All calculations were performed with spin polarization using only the Γ-point. Geometry optimizations were considered converged upon reaching a maximum force of less than 0.02 eV/Å per atom, with a self-consistent field (SCF) energy convergence threshold of 3 × 10^−5^ eV.

For the purpose of evaluating the reducibility of Sm-doped ceria, the oxygen vacancy formation energy ($E_{vac}$) was first computed for pristine ceria systems as the reference state. Subsequently, $E_{vac}$ in the Sm-doped systems, determined by removing an oxygen atom to form the reduced structure (defect, $E_{def}$) from the corresponding stoichiometric structure (perfect, $E_{perf}$), was calculated as follows:

(1) $$E_{vac}=E_{def}+\frac{1}{2}E_{O_{2}}-E_{perf}$$

In the expression above, $E_{O_{2}}$ refers to the total energy of the free diatomic oxygen molecule in its triplet ground state, calculated with the identical supercell parameters as those applied in the corresponding ceria system calculation. It is important to note that all the modeled systems are neutral.

### 3.2. CeO_2_(111) Slab Model

The periodic slab model, with the stoichiometry of Ce_64_O_128_ and containing four CeO_2_ tri-layers, was derived from an optimized bulk structure and has cell parameters of *a* = 15.486 Å, *b* = 15.486 Å, *c* = 21.064 Å, *α* = 90°, *β* = 90°, *γ* = 120°. During the geometry optimization of the periodic slab surface structures, all atomic positions were fully relaxed while the cell parameters were kept fixed.

### 3.3. Ce_140_O_280_ Nanoparticle Model

In line with experimental observations on the shapes of ceria nanoparticles [63], we selected a truncated octahedral Ce_140_O_280_ model with a diameter of ~2.4 nm [64], with the structural stability that has been established by previous theoretical calculations based on global optimization and density functional theory [59,64]. The simulation environment was set up by placing the nanoparticle model in a 32 × 32 × 32 Å^3^ cubic box, which provided an adequate vacuum spacing of about 8 Å between periodic images to prevent interparticle interactions.

## 4. Conclusions

In this study, DFT investigations were conducted to elucidate the effect of Sm ion doping on the structure, stability, and reducibility of ceria systems, with a focus on the periodic CeO_2_(111) slab and Ce_140_O_280_ nanoparticle models as two distinct morphologies. Among all studied structures, including the preferences for mono- (one Sm^3+^ ion) and bi-doping (two Sm^3+^ ions) in different atomic layers, substitution of Ce with Sm ion(s) in the ceria surface layer appeared to exhibit the most energetically favorable structures. This preference is likely attributed to the ionic radius of Sm^3+^ cations, which is by 10.9 pm larger than that of Ce^4+^ cations, resulting in steric hindrance in higher-coordinated subsurface sites. Furthermore, our analysis of bi-doped systems revealed that structures with the second Sm ion located close to the first demonstrated energetic stability equal or greater than arrangements where Sm ions are farther apart.

For the predicted most stable doped models, we studied and identified the most favorable sites for oxygen vacancy formation, finding that a clear trend in almost all selected Sm-doped CeO_2_(111) models: the second anionic layer of the slab model is the most preferred location for creating an oxygen defect, especially when the removed O ion is located close to the dopant(s), yielding the lowest oxygen vacancy formation energy. However, reflecting the general preference for vacancies to form near the dopant position, a different trend emerged in the considered models of the Ce_63_SmO_127_ and Ce_62_Sm_2_O_127_ systems, representing our doped nanoparticles, where the lowest oxygen vacancy formation energies were found when removing two-coordinated O anions directly bonded to the dopant(s). Overall, by evaluating and comparing these calculated oxygen vacancy formation energies, our findings indicate that Sm doping significantly improves the reducibility of representative ceria systems, aligning well with experimental observations.

## Figures and Tables

**Table 1:** Position of Sm ion(s) in the Sm-doped CeO_2_(111) surface, relative energy of the structures in these positions, ΔE, shortest distance between the two Sm^3+^ ions, d(Sm-Sm), the coordination number of Sm ion(s) with respect to the closest oxygen centers, CN, and the magnetic moment of substituted Sm ion(s), µ(Sm).

System	Position ^a^	ΔE/eV	d(Sm-Sm)/pm	CN	µ(Sm)/µB
Ce_63_SmO_128_	Surf	0.00	-	7	4.88
	Sub	0.09	-	8	4.82
Ce_62_Sm_2_O_128_	11_C	0.00	384	7, 7	4.89, 4.89
	11_F	0.07	774	7, 7	4.88, 4.88
	12_C	0.08	382	7, 8	4.88, 4.84
	12_F	0.15	670	7, 8	4.88, 4.82
	13_C	0.16	671	7, 8	4.88, 4.82
	13_F	0.16	866	7, 8	4.88, 4.82
	14_C	0.07	948	7, 7	4.88, 4.88
	14_F	0.07	1224	7, 7	4.88, 4.88
	22_C	0.13	384	8, 8	4.83, 4.83
	22_F	0.25	774	8, 8	4.83, 4.82
	23_C	0.14	386	8, 8	4.83, 4.83
	23_F	0.25	866	8, 8	4.83, 4.81

^a^ The notations “C” (close) or “F” (far) indicate the shortest and farthest distance between Sm^3+^ cations for the current combination of ceria layers. The shortest distance corresponds to two adjacent (neighboring) Sm^3+^, only when both cations are located in the same or neighboring layers.

**Table 2:** Ranges of oxygen vacancy formation energies, E_vac_ (Equation (1)), for different positions of O center on the stoichiometric Sm doped CeO_2_(111) surface (based on Figure 2), along with the corresponding relative values ΔE_vac_ with respect to the most stable reduced structure in each system. The numbers of reduced Ce^3+^ and Sm^3+^ cations are also shown. Results are reported for both Sm mono-doped CeO_2_(111) models and three selected Sm bi-doped CeO_2_(111) models. The results for pristine surface, included as a reference, are also presented. The number of considered configurations for each model is given in parentheses. Further details can be found in the ESI.

System	Model	E_vac_ Range/eV	ΔE_vac_ Range/eV	#Ce^3+^	#Sm^3+^
Ce_64_O_127_		2.43–3.25 (4)	0.00–0.82	2	-
Ce_63_SmO_127_	Surf	1.41–1.90 (6)	0.18–0.66	1	1
	Sub	1.24–2.04 (8)	0.00–0.81	1	1
Ce_62_Sm_2_O_127_	11_C	0.50–0.84 (6)	0.22–0.55	0	2
	11_F	0.45–0.80 (6)	0.17–0.52	0	2
	22_C	0.29–0.98 (6)	0.00–0.69	0	2

**Table 3:** Position of Sm ion(s) in the Sm-doped Ce_140_O_280_ nanoparticle, relative energy of the structures in these positions, ΔE, shortest distance between the two Sm^3+^ ions, d(Sm-Sm), the coordination number of Sm ion(s) with respect to the closest oxygen centers, CN, and the magnetic moment of substituted Sm ion(s), µ(Sm).

System	Position	ΔE/eV	d(Sm-Sm)/pm	CN	µ(Sm)/µB
Ce_139_SmO_280_	S_D	0.00	-	6	4.92
	S_A	0.07	-	6	4.94
	S_B	0.12	-	7	4.91
	S_C	0.12	-	7	4.91
	I_J	0.04	-	8	4.78
	I_K	0.14	-	8	4.83
	I_F	0.17	-	8	4.80
	I_I	0.17	-	8	4.85
Ce_138_Sm_2_O_280_	DD_C	0.00	374	6, 6	4.92, 4.93
	DD_F	0.19	2213	6, 6	4.92, 4.92
	DJ_C	0.07	377	6, 8	4.88, 4.83
	DJ_F	0.17	1890	6, 8	4.89, 4.81

**Table 4:** Ranges of oxygen vacancy formation energies, E_vac_ (Equation (1)), for different positions of O-atom on the stoichiometric Sm-doped Ce_140_O_280_ nanoparticle (based on Figure 4 and Figure 5), along with the corresponding relative values ΔE_vac_ with respect to the most stable reduced structure in each system and the numbers of reduced Ce^3+^ and Sm^3+^ cations. Results are reported for the two most stable Sm mono-doped nanoparticle models and the four considered Sm bi-doped nanoparticle models, with results for pristine nanoparticle included as a reference. The number of considered configurations for each model is given in parentheses. Further details are found in the ESI.

System	Model	E_vac_ Range/eV	ΔE_vac_ Range/eV	#Ce^3+^	#Sm^3+^
Ce_140_O_279_		1.04–2.22 (3)	0.00–1.18	2	-
Ce_139_SmO_279_	S_D	0.34–1.99 (16)	0.10–1.74	1	1
	I_J	0.25–1.71 (6)	0.00–1.46	1	1
Ce_138_Sm_2_O_279_	DD_C	−0.10–1.81 (8)	0.11–2.01	0 or 1	2
	DD_F	0.09–1.65 (5)	0.30–1.86	0 or 1	2
	DJ_C	−0.21–1.71 (10)	0.00–1.92	0 or 1	2
	DJ_F	−0.06–1.21 (10)	0.14–1.42	0	2

## Data Availability

The original contributions presented in this study are included in the article/Supplementary material. Further inquiries can be directed to the corresponding author(s).

## Supplementary Materials

The following supporting information can be downloaded at: https://www.mdpi.com/article/10.3390/molecules30234615/s1, Table S1. Oxygen vacancy formation energy information for Sm doped CeO_2_(111) surface. Table S2. Oxygen vacancy formation energy information for Sm doped Ce_140_O_280_ nanoparticle.
