# Supplementary material for: Impact of Sm3+ Ions on Oxygen Vacancy Formation in Ceria Systems
Source: Molecules. 2025 Dec 1;30(23):4615. doi: 10.3390/molecules30234615 (PMC12693117; doi:10.3390/molecules30234615)
Supplement: Supplementary file 1 [file molecules-30-04615-s001.zip › molecules-3999760-supplementary.pdf]

Contents

Table S1. Oxygen vacancy formation energy information for Sm doped CeO_2(111) surface.

Table S2. Oxygen vacancy formation energy information for Sm doped $Ce_{140}O_{280}$ nanoparticle.

Table S1. Oxygen vacancy formation energies, E_{vac}, for different O-atom positions on the stoichiometric pristine and Sm doped $CeO_2(111)$ surface, the corresponding relative values ΔE_{vac} with respect to the most stable reduced structure in each system, distance between the Sm cation and the position of removed O, d(Sm-O_{vac}), and the numbers of reduced Ce^{3+} and Sm^{3+}cations, #Ce^{3+} and #Sm^{3+}, respectively. Results are reported for both Sm mono-doped $CeO_2(111)$ models and three selected Sm bi-doped $CeO_2(111)$ models, as well as for pristine surface included as a reference. The highlighted rows in bold represent the most stable reduced structure for each system.

System	*Model*	*Position of O_{vac}*	E_{vac}/eV	$\Delta E_{vac}/eV$	*d(Sm-O_{vac})/pm*	*#Ce^{3+}*	*#Sm^{3+}*
$Ce_{64}O_{127}$		O3_1st	2.57	0.14	-	2	-
		**O4_2nd**	**2.43**	**0.00**	**-**	**2**	**-**
		O4_3rd	2.74	0.31	-	2	-
		O4_4th	3.25	0.82	-	2	-
$Ce_{63}SmO_{127}$	Surf	O3_1st_C	1.46	0.22	239	1	1
	Surf	O3_1st_F	1.59	0.35	810	1	1
	Surf	O4_2nd_C	1.45	0.22	235	1	1
	Surf	O4_2nd_F	1.41	0.18	898	1	1
	Surf	O4_3rd_C	1.75	0.51	239	1	1
	Surf	O4_3rd_F	1.90	0.66	710	1	1
	Sub	O3_1st_C	1.42	0.18	452	1	1
	Sub	O3_1st_F	1.42	0.18	896	1	1
	Sub	**O4_2nd_C**	**1.24**	**0.00**	**235**	**1**	**1**
	Sub	O4_2nd_F	1.39	0.16	809	1	1
	Sub	O4_3rd_C	1.66	0.43	238	1	1
	Sub	O4_3rd_F	1.71	0.47	898	1	1
	Sub	O4_4th_C	1.90	0.66	238	1	1
	Sub	O4_4th_F	2.04	0.81	810	1	1
$Ce_{62}Sm_2O_{127}$	11_C	O3_1st_C	0.50	0.22	236, 236	0	2
	11_C	O3_1st_F	0.83	0.55	579, 896	0	2
	11_C	O4_2nd_C	0.55	0.27	234, 234	0	2
	11_C	O4_2nd_F	0.58	0.30	810, 810	0	2
	11_C	O4_3rd_C	0.84	0.55	239, 453	0	2
	11_C	O4_3rd_F	0.83	0.54	712, 1050	0	2
	11_F	O3_1st_C	0.55	0.27	240, 594	0	2
	11_F	O3_1st_F	0.68	0.39	596, 810	0	2
	11_F	O4_2nd_C	0.45	0.17	234, 599	0	2

11_F	O4_2nd_F	0.48	0.20	597, 810	0	2
11_F	O4_3rd_C	0.80	0.51	240, 811	0	2
11_F	O4_3rd_F	0.80	0.52	809, 809	0	2
22_C	**O4_2nd_C**	**0.29**	**0.00**	**235, 451**	**0**	**2**
22_C	O4_2nd_F	0.48	0.19	711, 808	0	2
22_C	O4_3rd_C	0.55	0.26	236, 236	0	2
22_C	O4_3rd_F	0.73	0.44	810, 810	0	2
22_C	O4_4th_C	0.74	0.45	235, 235	0	2
22_C	O4_4th_F	0.98	0.69	809, 809	0	2

Table S2. Oxygen vacancy formation energies, E_{vac}, for different O-atom positions on the stoichiometric pristine and Sm-doped $Ce_{140}O_{280}$ nanoparticle, the corresponding relative values ΔE_{vac} with respect to the most stable reduced structure in each system, distance between the Sm cation and the position of removed O, d(Sm-O_{vac}), and the numbers of reduced Ce^{3+} and Sm^{3+}cations, #Ce^{3+} and #Sm^{3+}, respectively. Results are reported for the two most stable Sm mono-doped nanoparticle models and the four considered Sm bi-doped nanoparticle models, as well for pristine surface included as a reference. The highlighted rows in bold represent the most stable reduced structure for each system.

System	*Model*	*Position of O_{vac}*	E_{vac}/eV	$\Delta E_{vac}/eV$	*d(Sm-O_{vac})/pm*	*#Ce^{3+}*	*#Sm^{3+}*
$Ce_{140}O_{279}$		**O2**	**1.04**	**0.00**	**-**	**2**	**-**
		O3	2.22	1.18	-	2	-
		O4	1.04	0.00	-	2	-
$Ce_{139}SmO_{279}$	S_D	O2_C1	0.48	0.23	218	1	1
	S_D	O2_F1	0.78	0.83	2266	1	1
	S_D	O2_C2	0.62	0.38	464	1	1
	S_D	O2_F2	0.92	0.67	2303	1	1
	S_D	O3_C1	1.76	1.51	243	1	1
	S_D	O3_F1	1.95	1.71	2026	1	1
	S_D	O3_C2	1.65	1.41	451	1	1
	S_D	O3_F2	1.97	1.72	2061	1	1
	S_D	O3_C3	1.77	1.52	701	1	1
	S_D	O3_F3	1.99	1.74	2130	1	1
	S_D	O3_C4	1.75	1.51	590	1	1
	S_D	O3_F4	1.96	1.71	2096	1	1
	S_D	O4_C1	0.34	0.10	236	1	1
	S_D	O4_F1	0.89	0.65	2015	1	1
	S_D	O4_C2	0.39	0.14	443	1	1
	S_D	O4_F2	0.89	0.64	2049	1	1
	I_J	O2_C	0.56	0.31	445	1	1
	I_J	O2_F	0.81	0.56	1995	1	1
	I_J	O3_C	1.41	1.17	456	1	1
	I_J	O3_F	1.71	1.46	1794	1	1
	I_J	**O4_C**	**0.25**	**0.00**	**230**	**1**	**1**
	I_J	O4_F	0.81	0.56	1741	1	1
$Ce_{138}Sm_2O_{279}$	DD_C	O2_C1	-0.09	0.11	220, 219	0	2
	DD_C	O2_C2	0.09	0.30	465, 219	0	2
	DD_C	O2_C3	0.21	0.42	463, 463	0	2
	DD_C	O2_F	0.60	0.81	2264, 2263	1	2

DD_C	O3_C	1.58	1.78	242, 452	1	2
DD_C	O3_F	1.81	2.01	2023, 2059	1	2
DD_C	O4_C	-0.10	0.11	234, 234	0	2
DD_C	O4_F	0.74	0.94	2012, 2012	1	2
DD_F	O2_1	0.09	0.30	218, 2302	0	2
DD_F	O2_2	0.23	0.43	464, 2267	0	2
DD_F	O3_1	1.65	1.86	590, 2061	1	2
DD_F	O3_2	1.36	1.56	243, 2130	0	2
DD_F	O4	0.10	0.30	236, 2050	0	2
DJ_C	**O2_C1**	**-0.20**	**0.00**	**217, 432**	**0**	**2**
DJ_C	O2_F1	0.81	1.01	2262, 1996	1	2
DJ_C	O2_C2	-0.08	0.12	463, 444	0	2
DJ_C	O2_F2	0.72	0.92	2299, 1997	1	2
DJ_C	O3_C1	1.12	1.32	241, 455	0	2
DJ_C	O3_C2	1.15	1.36	699, 457	0	2
DJ_C	O3_F	1.71	1.92	2022, 1794	1	2
DJ_C	O4_C1	-0.10	0.11	442, 231	0	2
DJ_C	O4_C2	-0.21	0.00	234, 231	0	2
DJ_C	O4_F	0.54	0.74	2011, 1742	1	2
DJ_F	O2_1	-0.06	0.14	217, 1994	0	2
DJ_F	O2_2	0.07	0.27	464, 1995	0	2
DJ_F	O2_3	0.15	0.36	2264, 445	0	2
DJ_F	O3_1	1.21	1.42	241, 1793	0	2
DJ_F	O3_2	1.20	1.40	450, 1794	0	2
DJ_F	O3_3	1.20	1.40	590, 1794	0	2
DJ_F	O3_4	1.18	1.38	2130, 457	0	2
DJ_F	O4_1	-0.05	0.15	236, 1742	0	2
DJ_F	O4_2	0.07	0.27	444, 1740	0	2
DJ_F	O4_3	0.15	0.35	2048, 231	0	2
